# Supplementary figures and images for: Using an Antibiogram Profile to Improve Infection Control and Rational Antimicrobial Therapy in an Urban Hospital in The Gambia, Strategies and Lessons for Low- and Middle-Income Countries
Source: Antibiotics (Basel). 2023 Apr 21;12(4):790. doi: 10.3390/antibiotics12040790 (PMC10135392; doi:10.3390/antibiotics12040790)

Suppl figure S1a

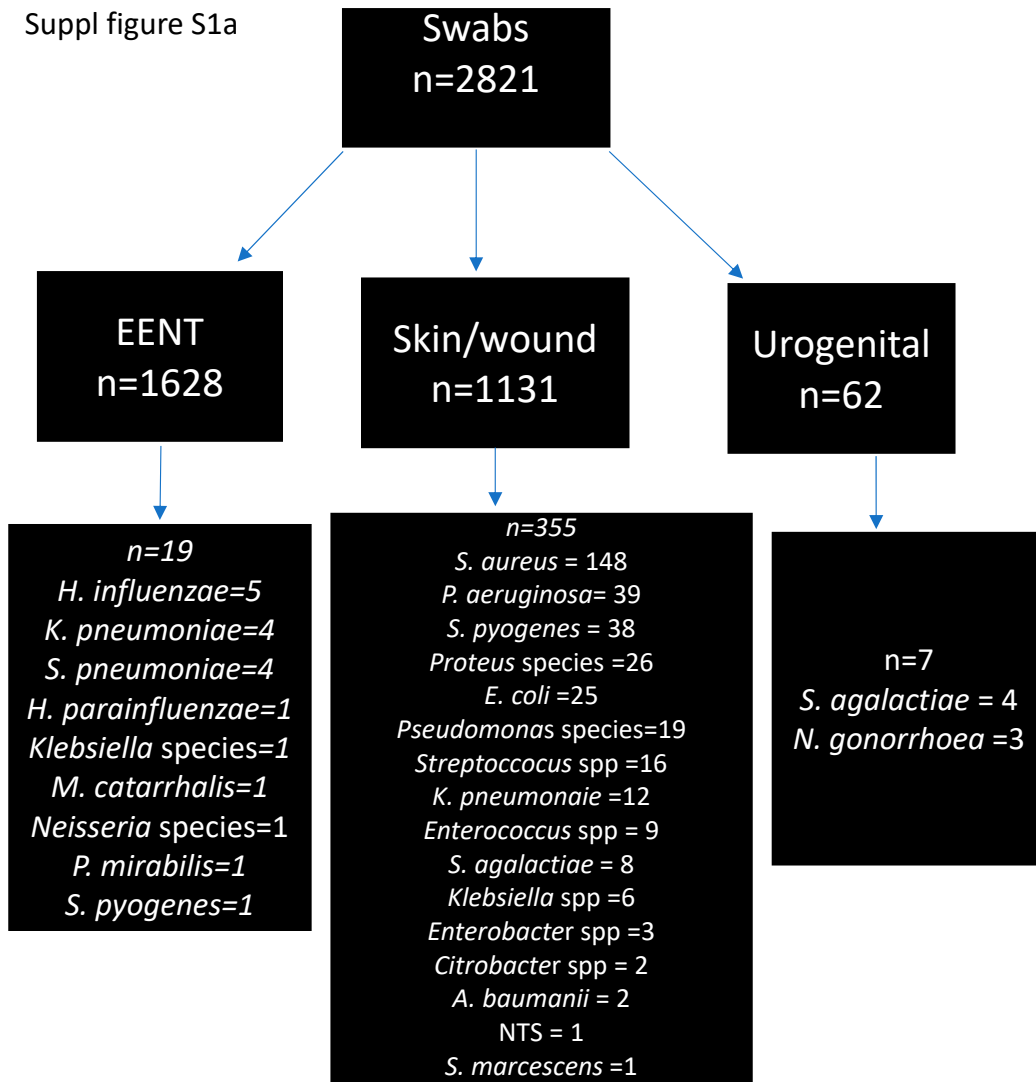

Suppl figure S1b

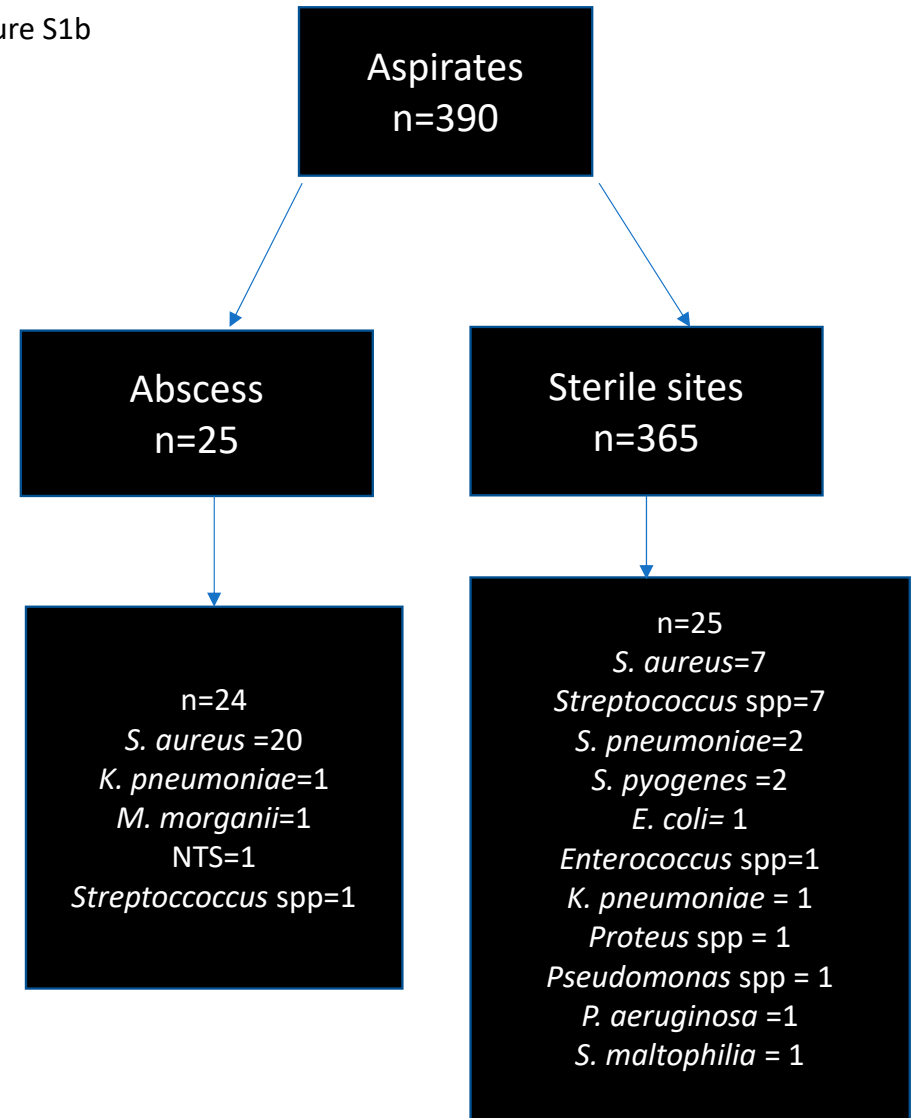

Supplement: Supplementary file 1 [file antibiotics-12-00790-s001.zip › Supple Figures_ S1.pdf]

Supplementary figure S2

OVERALL DISTRIBUTION OF PATHOGENS AMONG DIFFERENT SAMPLES

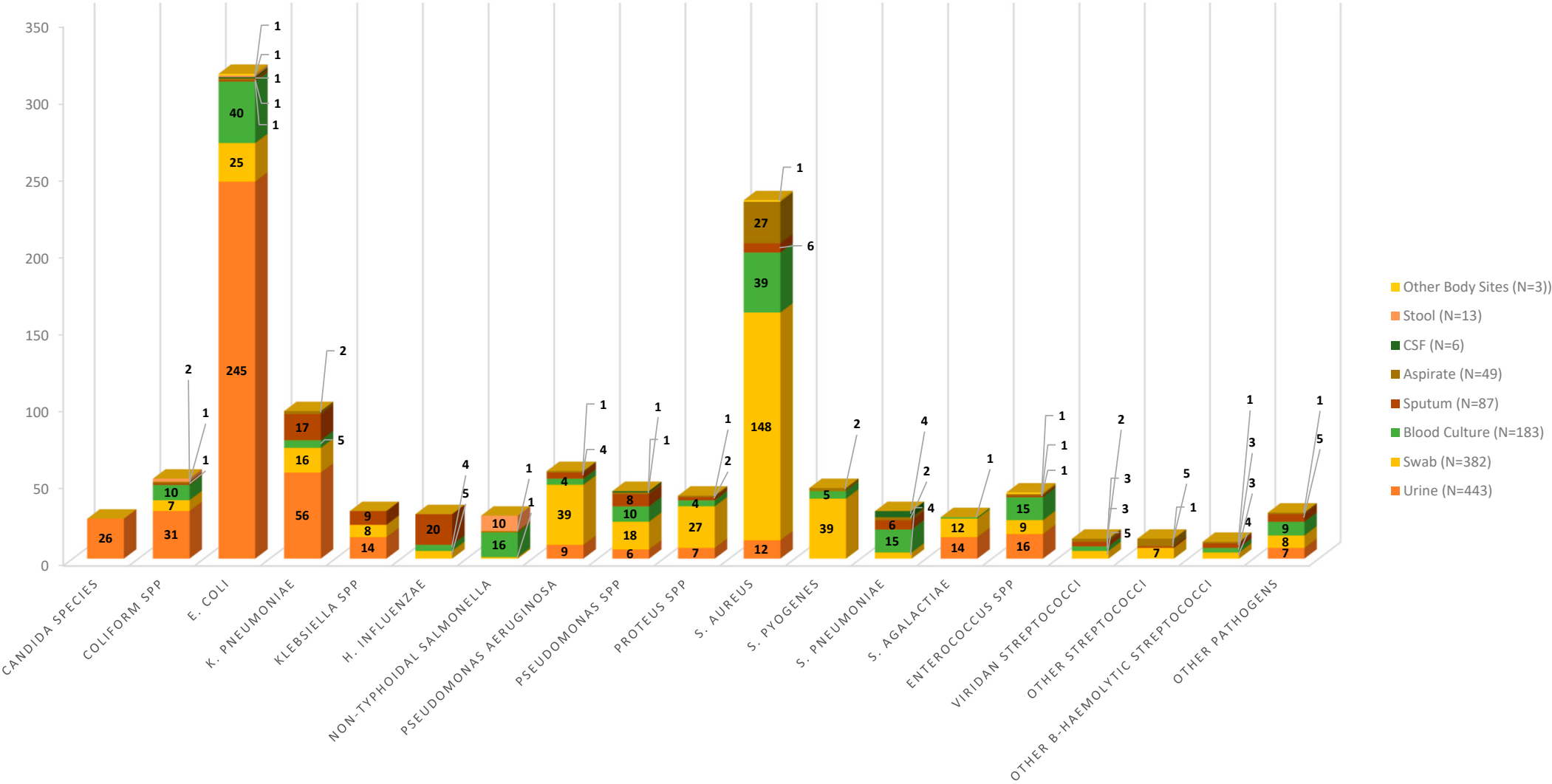

Supplement: Supplementary file 1 [file antibiotics-12-00790-s001.zip › Supplemenatary fig S2.pdf]
